# Tracing the first steps of American sturgeon pioneers in Europe

**DOI:** 10.1186/1471-2148-8-221

**Published:** 2008-07-29

**Authors:** Arne Ludwig, Ursula Arndt, Sebastian Lippold, Norbert Benecke, Lutz Debus, Timothy L King, Shuichi Matsumura

**Affiliations:** 1 Leibniz Institute for Zoo and Wildlife Research, Evolutionary Genetics, 12561 Berlin, Germany; 2 Palaeogenetics Laboratory, Johannes Gutenberg University, 55118 Mainz, Germany; 3 Simon Fraser University, Department for Archaeology, Burnaby, BC, Canada; 4 German Archaeological Institute, Department of Eurasia, Im Dol 2-6, 14195 Berlin, Germany; 5 Aquaculture, Forstweg 1, 31582 Nienburg/Weser, Germany; 6 United States Geological Survey, Leetown Science Center, 11649 Leetown Road, Kearneysville, West Virginia, 25430, USA; 7 International Institute for Applied Systems Analysis, A-2361 Laxenburg, Austria; 8 Leibniz-Institute of Freshwater Ecology and Inland Fisheries, 12567 Berlin, Germany

## Abstract

**Background:**

A Baltic population of Atlantic sturgeon was founded ~1,200 years ago by migrants from North America, but after centuries of persistence, the population was extirpated in the 1960s, mainly as a result of over-harvest and habitat alterations. As there are four genetically distinct groups of Atlantic sturgeon inhabiting North American rivers today, we investigated the genetic provenance of the historic Baltic population by ancient DNA analyses using mitochondrial and nuclear markers.

**Results:**

The phylogeographic signal obtained from multilocus microsatellite DNA genotypes and mitochondrial DNA control region haplotypes, when compared to existing baseline datasets from extant populations, allowed for the identification of the region-of-origin of the North American Atlantic sturgeon founders. Moreover, statistical and simulation analyses of the multilocus genotypes allowed for the calculation of the effective number of individuals that originally founded the European population of Atlantic sturgeon. Our findings suggest that the Baltic population of *A. oxyrinchus *descended from a relatively small number of founders originating from the northern extent of the species' range in North America.

**Conclusion:**

These results demonstrate that the most northerly distributed North American *A. oxyrinchus *colonized the Baltic Sea ~1,200 years ago, suggesting that Canadian specimens should be the primary source of broodstock used for restoration in Baltic rivers. This study illustrates the great potential of patterns obtained from ancient DNA to identify population-of-origin to investigate historic genotype structure of extinct populations.

## Background

Sturgeons (Acipenseriformes: Acipenseridae), the producers of caviar, are remnant survivors of the once flourishing chondrosteans, dominant fishes of the Permian period. The continued persistence of these 'living fossils' is threatened throughout North America, Europe, and Asia. Today there are two species of Atlantic sea sturgeons; the European sturgeon *Acipenser sturio*, found in France (Gironde basin), and the Atlantic sturgeon *A. oxyrinchus *inhabiting the rivers and coastal waters from the Gulf of Mexico to the Canadian Maritime Provinces. Although classified as sister species and showing some phenotypic similarities, approximately 60 million years of isolation [1] has resulted in physiological differences between these two species. For example, European sturgeons prefer spawning temperatures ≥ 20°C, while Atlantic sturgeons exhibit latitudinal variation in spawning temperatures ranging from as low as 13°C in Canada to 26°C in the southeastern U.S. [2].

According to archaeological and molecular dating, a population of Atlantic sturgeon was founded in the Baltic Sea during the Middle Ages (8^th ^and 10^th ^century) by migrants from North America [3]. These founders created a self-sustaining population, which became disjunct from the western Atlantic populations. This Baltic population has been over-exploited by commercial fisheries and was extirpated in the 20^th ^century. A group of international fishery managers are now seeking to re-establish the extirpated population using fish from the original source population(s), on the grounds that North American *A. oxyrinchus *exhibit sufficient ecological and genetic potential for a successful restoration. To increase the probability of success of such a restoration in the long-term, the ideal scenario would be to identify and use a founder group that is genetically closely related to the extinct population. Although the utility of ancient DNA studies to elucidate evolutionary relationships and guide restoration projects has been recognized [4-7], the full extent of management applications from these studies have not yet been realized.

In this study, we investigated the evolutionary and demographic characteristics of the historic founders, by performing an extensive genetic characterization of the extinct Baltic population derived from medieval tissue samples representing their first generations starting at the 8^th ^century. We focused on identifying the region-of-origin of the North American founders, and on calculating the effective number of individuals that originally founded the Baltic population ~1,200 years ago.

## Results

### Mitochondrial DNA (mtDNA)

Two hundred and twenty seven DNA samples from 586 ancient bony scutes (8^th ^– 13^th ^c.) were successfully screened for their mtDNA control region haplotypes. The species *A. sturio *and *A. oxyrinchus *were differentiated by 22 diagnostic substitutions (> 10% sequence divergence) [see Additional file 1]. Two hundred and twenty scutes had *A. oxyrinchus *control region haplotypes (218 haplotype A, and one haplotype BS1 [EU684143] and BS2 [EU684144] each, respectively). Seven scutes shared haplotype AS17 from *A. sturio*.

### Morphological classification

The morphology of 210 bony scutes was preserved sufficiently to identify species. Of this number, 176 were classified as *A. oxyrinchus*; whereas 34 showed typical *A. sturio *surfaces. Morphological classifications were subject to error depending on the state of scute preservation. However, 183 (87%) samples were classified as the same species based on morphology and mitochondrial DNA. Four scutes yielding *A. sturio *haplotypes showed *A. oxyrinchus *morphology; in contrast 23 scutes had *A. oxyrinchus *mtDNA and *A. sturio *morphology.

### Amplification of nuclear DNA

**Figure 1:**
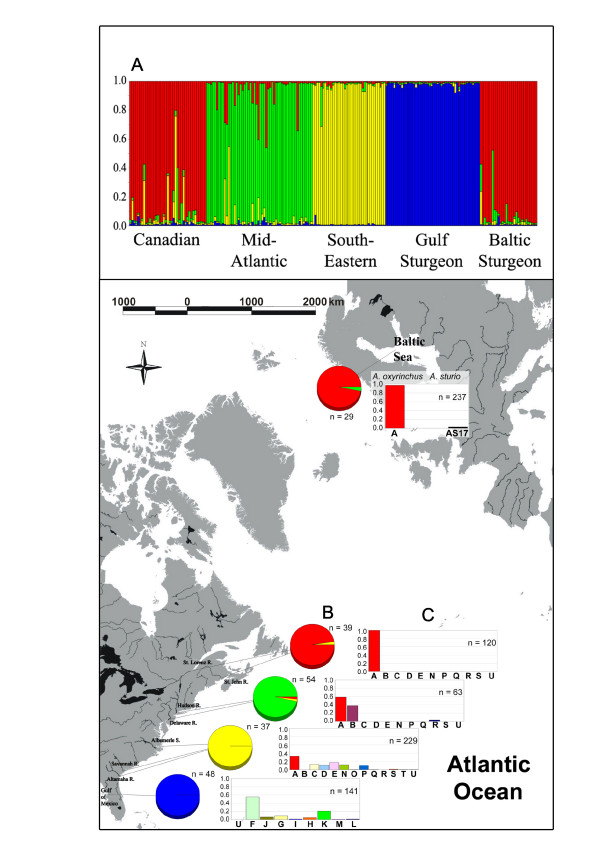
**Genetic variation and assignment test**. A) Assignment test conducted in STRUCTURE based on seven polymorphic microsatellites showing Atlantic sturgeon genotype structuring and the assignment of Baltic individuals; B) Pie charts are the frequencies of the assignment to each sub-population calculated in STRUCTURE. Colors are identical with the population subdivision observed in the assignment test A; C) Histograms illustrates mitochondrial haplotype frequencies from each locality. Baltic sturgeon data were taken from this study (n = 227 ancient DNA samples) and 10 archived specimens previously published [3], Atlantic sturgeon data from 3, 9 and Gulf sturgeon *A. oxyrinchus desotoi *were published by 10.

Allelic profiles of 29 (out of 50) randomly selected scutes from Ralswieck, Island of Rugia Germany were successfully amplified. The 29 randomly selected scutes yielded unique multilocus genotypes. Locus *Afu-39 *was monomorphic in two populations (Table 1). Profiles of seven polymorphic microsatellite loci were used for the assignment analysis: *Afu-19 *(trinucleotide), *Afu-39 *(trinucleotide), *Afu-68 *(tetranucleotide), *Afu-54 *(tetranucleotide), *Aox-45 *(trinucleotide), *Aox-23 *(trinucleotide) and *Aox-12 *(imperfect nucleotide). All loci used in this study showed allelic patterns of disomic inheritance. The detected structure (four clusters) of *A. oxyrinchus *populations was related to their geographic distribution. Baltic and Canadian sturgeons grouped together (Figure 1A). STRUCTURE results showed a high allele-frequency similarity of Baltic samples with Canadian samples (28 samples were assigned to the Canadian population). A single sample was assigned to the Mid-Atlantic population. Probability values for region-of-origin assignment are given in Table 2. F_ST _estimates (10100 permutations) (Table 3) and AMOVA values (Table 4) were calculated using Arlequin v. 3.0 [8] based on haplotype frequencies of mtDNA control region sequences.

**Table 1:** Heterozygosity.

Locus	n	H_o_	H_e_	*p*	s.d.	Steps done
Canadian population						

*Afu19*	39	0.31	0.33	1.00	0	10100
*Afu39*	39	This locus is monomorphic: no test done.				
*Afu54*	39	0.26	0.28	1.00	0	10100
*Afu68*	39	0.49	0.49	0.34	> 0	10100
*Aox23*	39	0.56	0.58	0.28	> 0	10100
*Aox45*	39	0.72	0.77	0.83	> 0	10100
*Aox12*	39	0.77	0.83	0.01	> 0	10100

Mid-Atlantic population						

*Afu19*	54	0.74	0.65	0.01	> 0	10100
*Afu39*	54	0.04	0.05	1	0	10100
*Afu54*	54	0.35	0.31	0.68	> 0	10100
*Afu68*	54	0.72	0.75	0.63	> 0	10100
*Aox23*	54	0.76	0.69	0.61	> 0	10100
*Aox45*	54	0.85	0.85	0.08	> 0	10100
*Aox12*	53	0.85	0.84	0	0	10100

Southeast population						

*Afu19*	37	0.67	0.65	0.59	> 0	10100
*Afu39*	37	0.38	0.33	0.31	> 0	10100
*Afu54*	37	0.27	0.24	1	0	10100
*Afu68*	37	0.81	0.80	0.39	> 0	10100
*Aox23*	37	0.70	0.75	0.87	> 0	10100
*Aox45*	37	0.78	0.82	0.02	> 0	10100
*Aox12*	37	0.84	0.84	0.07	> 0	10100

Gulf population						

*Afu19*	48	0.58	0.59	0.59	> 0	10100
*Afu39*	48	0.35	0.31	0.32	> 0	10100
*Afu54*	48	0.54	0.51	0.56	> 0	10100
*Afu68*	48	0.23	0.23	0.35	> 0	10100
*Aox23*	48	0.33	0.40	0.53	> 0	10100
*Aox45*	48	0.73	0.67	0.68	> 0	10100
*Aox12*	48	0.73	0.72	> 0	> 0	10100

Baltic population (aDNA)						

*Afu19*	24	0.56	0.67	0.29	> 0	10100
*Afu39*	18	This locus is monomorphic: no test done.				
*Afu54*	29	0.29	0.42	0.13	> 0	10100
*Afu68*	29	0.54	0.73	0.25	> 0	10100
*Aox23*	26	0.52	0.67	> 0	> 0	10100
*Aox45*	29	0.61	0.76	0.07	> 0	10100
*Aox12*	30	0.53	0.65	0.07	> 0	10100

Heterozygosity based on microsatellites calculated in Arlequin v. 3.0 [8,40].

**Table 2:** Probability of assignment values.

Sample	Cluster 1	Cluster 2	Cluster 3	Cluster 4
Canadian_01	**0.960**	0.021	0.006	0.013
Canadian_02	**0.803**	0.024	0.135	0.038
Canadian_03	**0.963**	0.011	0.009	0.017
Canadian_04	**0.970**	0.017	0.007	0.005
Canadian_05	**0.913**	0.013	0.010	0.064
Canadian_06	**0.982**	0.007	0.007	0.004
Canadian_07	**0.951**	0.018	0.026	0.006
Canadian_08	**0.575**	0.113	0.307	0.005
Canadian_09	**0.982**	0.005	0.006	0.006
Canadian_10	**0.978**	0.013	0.006	0.004
Canadian_11	**0.947**	0.033	0.015	0.004
Canadian_12	**0.980**	0.008	0.007	0.005
Canadian_13	**0.954**	0.010	0.029	0.007
Canadian_14	**0.930**	0.026	0.034	0.010
Canadian_15	**0.928**	0.043	0.02	0.009
Canadian_16	**0.981**	0.006	0.007	0.006
Canadian_18	**0.975**	0.011	0.005	0.009
Canadian_19	**0.918**	0.020	0.056	0.007
Canadian_20	**0.960**	0.017	0.006	0.017
Canadian_41	**0.636**	0.017	0.339	0.008
Canadian_42	**0.964**	0.015	0.015	0.006
Canadian_43	**0.966**	0.014	0.005	0.014
Canadian_44	**0.757**	0.078	0.112	0.053
Canadian_45	0.202	0.039	**0.742**	0.017
Canadian_46	**0.599**	0.371	0.024	0.006
Canadian_47	**0.962**	0.018	0.012	0.009
Canadian_48	**0.844**	0.145	0.007	0.004
Canadian_49	**0.613**	0.047	0.304	0.036
Canadian_50	**0.973**	0.008	0.011	0.007
Canadian_51	**0.937**	0.025	0.014	0.024
Canadian_52	**0.898**	0.071	0.022	0.008
Canadian_53	**0.951**	0.012	0.010	0.028
Canadian_54	**0.896**	0.065	0.025	0.014
Canadian_55	**0.892**	0.033	0.068	0.007
Canadian_56	**0.977**	0.011	0.007	0.006
Canadian_57	**0.976**	0.012	0.007	0.006
Canadian_58	**0.970**	0.012	0.012	0.005
Canadian_59	**0.974**	0.009	0.006	0.011
Canadian_60	**0.980**	0.006	0.008	0.006

Mid-American_01	0.011	**0.973**	0.008	0.008
Mid-American_02	0.015	**0.971**	0.008	0.006
Mid-American_03	0.023	**0.951**	0.020	0.005
Mid-American_04	0.006	**0.972**	0.017	0.004
Mid-American_05	0.006	**0.932**	0.029	0.033
Mid-American_06	0.198	**0.775**	0.010	0.018
Mid-American_07	0.006	**0.966**	0.008	0.020
Mid-American_08	0.007	**0.984**	0.005	0.004
Mid-American_09	0.007	**0.977**	0.004	0.012
Mid-American_10	0.286	**0.39**	0.315	0.009
Mid-American_11	0.299	**0.635**	0.062	0.004
Mid-American_13	0.015	0.435	**0.545**	0.005
Mid-American_14	0.017	**0.972**	0.006	0.005
Mid-American_15	0.004	**0.988**	0.005	0.004
Mid-American_16	0.169	**0.685**	0.137	0.008
Mid-American_17	0.008	**0.978**	0.010	0.004
Mid-American_18	0.094	**0.851**	0.017	0.039
Mid-American_19	0.020	**0.962**	0.009	0.009
Mid-American_20	0.064	**0.905**	0.027	0.004
Mid-American_21	0.019	**0.952**	0.019	0.01
Mid-American_22	0.006	**0.984**	0.007	0.003
Mid-American_23	0.059	**0.916**	0.01	0.014
Mid-American_24	0.007	**0.975**	0.015	0.003
Mid-American_25	0.151	**0.695**	0.129	0.025
Mid-American_26	0.008	**0.983**	0.005	0.003
Mid-American_27	0.160	**0.829**	0.006	0.005
Mid-American_28	**0.405**	0.403	0.158	0.035
Mid-American_29	0.015	**0.969**	0.011	0.005
Mid-American_31	0.017	**0.903**	0.045	0.035
Mid_American_97_01	0.016	**0.862**	0.065	0.057
Mid_American_97_02	0.459	**0.505**	0.013	0.023
Mid_American_97_03	0.052	**0.932**	0.013	0.004
Mid_American_97_04	0.043	**0.926**	0.013	0.018
Mid_American_97_05	0.005	**0.981**	0.009	0.005
Mid_American_97_06	0.159	**0.814**	0.024	0.003
Mid_American_97_07	0.007	**0.944**	0.044	0.004
Mid_American_97_08	0.008	**0.974**	0.005	0.013
Mid_American_97_09	0.005	**0.966**	0.016	0.013
Mid_American_97_10	0.016	**0.970**	0.008	0.006
Mid_American_97_11	0.019	**0.970**	0.008	0.003
Mid_American_97_12	0.027	**0.960**	0.007	0.006
Mid_American_97_13	0.020	**0.961**	0.015	0.003
Mid_American_97_14	0.006	**0.978**	0.010	0.005
Mid_American_97_15	0.012	**0.975**	0.008	0.004
Mid_American_97_16	0.012	**0.923**	0.060	0.006
Mid_American_97_17	0.008	**0.976**	0.008	0.008
Mid_American_97_18	0.343	**0.643**	0.008	0.006
Mid_American_97_19	0.016	**0.942**	0.012	0.030
Mid_American_97_20	0.009	**0.935**	0.052	0.005
Mid_American_97_21	0.016	**0.954**	0.018	0.012
Mid_American_97_22	0.011	**0.93**	0.033	0.027
Mid_American_97_23	0.018	**0.955**	0.005	0.022
Mid_American_97_24	0.009	**0.978**	0.007	0.007
Mid_American_97_25	0.015	**0.958**	0.018	0.009

South-East_01	0.015	0.008	**0.971**	0.007
South-East_02	0.023	0.008	**0.898**	0.071
South-East_03	0.005	0.006	**0.981**	0.008
South-East_04	0.006	0.005	**0.98**	0.008
South-East_05	0.037	0.280	**0.677**	0.006
South-East_06	0.007	0.041	**0.947**	0.005
South-East_07	0.005	0.007	**0.984**	0.004
South-East_08	0.036	0.018	**0.939**	0.006
South-East_09	0.023	0.009	**0.962**	0.006
South-East_10	0.048	0.013	**0.928**	0.011
South-East_11	0.010	0.017	**0.966**	0.008
South-East_12	0.006	0.007	**0.982**	0.005
South-East_13	0.007	0.006	**0.981**	0.005
South-East_14	0.005	0.007	**0.984**	0.004
South-East_15	0.009	0.006	**0.978**	0.007
South-East_16	0.008	0.006	**0.98**	0.005
South-East_17	0.020	0.032	**0.944**	0.004
South-East_18	0.013	0.012	**0.968**	0.007
South-East_19	0.007	0.007	**0.983**	0.004
South-East_20	0.007	0.013	**0.975**	0.005
South-East_21	0.007	0.008	**0.978**	0.006
South-East_22	0.011	0.008	**0.973**	0.008
South-East_23	0.008	0.011	**0.975**	0.006
South-East_24	0.007	0.008	**0.975**	0.01
South-East_25	0.010	0.008	**0.979**	0.004
South-East_26	0.048	0.024	**0.920**	0.008
South-East_27	0.006	0.066	**0.923**	0.005
South-East_28	0.005	0.005	**0.985**	0.005
South-East_29	0.013	0.007	**0.965**	0.015
South-East_30	0.008	0.009	**0.977**	0.005
South-East_31	0.023	0.009	**0.953**	0.015
South-East_32	0.021	0.014	**0.956**	0.009
South-East_33	0.007	0.005	**0.983**	0.004
South-East_34	0.004	0.004	**0.985**	0.006
South-East_35	0.034	0.009	**0.951**	0.006
South-East_36	0.005	0.013	**0.975**	0.006
South-East_37	0.004	0.008	**0.984**	0.004

Gulf sturgeon_073	0.018	0.008	0.005	**0.969**
Gulf sturgeon_074	0.005	0.003	0.004	**0.988**
Gulf sturgeon_075	0.005	0.004	0.005	**0.986**
Gulf sturgeon_076	0.005	0.004	0.004	**0.987**
Gulf sturgeon_077	0.009	0.013	0.026	**0.953**
Gulf sturgeon_078	0.004	0.004	0.004	**0.988**
Gulf sturgeon_079	0.006	0.006	0.005	**0.983**
Gulf sturgeon_080	0.012	0.006	0.006	**0.977**
Gulf sturgeon_081	0.008	0.016	0.024	**0.953**
Gulf sturgeon_082	0.008	0.006	0.006	**0.981**
Gulf sturgeon_083	0.004	0.009	0.007	**0.980**
Gulf sturgeon_084	0.004	0.004	0.005	**0.988**
Gulf sturgeon_085	0.005	0.005	0.004	**0.986**
Gulf sturgeon_086	0.005	0.005	0.005	**0.986**
Gulf sturgeon_087	0.004	0.007	0.007	**0.982**
Gulf sturgeon_088	0.010	0.009	0.026	**0.955**
Gulf sturgeon_089	0.005	0.005	0.006	**0.985**
Gulf sturgeon_090	0.005	0.014	0.007	**0.974**
Gulf sturgeon_091	0.008	0.007	0.007	**0.978**
Gulf sturgeon_092	0.006	0.005	0.005	**0.984**
Gulf sturgeon_093	0.007	0.007	0.005	**0.981**
Gulf sturgeon_094	0.005	0.006	0.007	**0.982**
Gulf sturgeon_095	0.013	0.016	0.006	**0.965**
Gulf sturgeon_096	0.011	0.009	0.014	**0.967**
Gulf sturgeon_137	0.006	0.004	0.005	**0.984**
Gulf sturgeon_138	0.004	0.006	0.005	**0.985**
Gulf sturgeon_139	0.007	0.006	0.006	**0.980**
Gulf sturgeon_140	0.005	0.004	0.004	**0.987**
Gulf sturgeon_141	0.017	0.012	0.005	**0.965**
Gulf sturgeon_142	0.007	0.006	0.007	**0.98**
Gulf sturgeon_143	0.006	0.004	0.005	**0.985**
Gulf sturgeon_144	0.005	0.005	0.005	**0.985**
Gulf sturgeon_145	0.006	0.005	0.005	**0.984**
Gulf sturgeon_146	0.007	0.006	0.007	**0.980**
Gulf sturgeon_147	0.006	0.007	0.025	**0.962**
Gulf sturgeon_148	0.009	0.009	0.065	**0.917**
Gulf sturgeon_149	0.004	0.005	0.004	**0.987**
Gulf sturgeon_150	0.037	0.017	0.018	**0.929**
Gulf sturgeon_151	0.009	0.008	0.006	**0.977**
Gulf sturgeon_152	0.009	0.006	0.007	**0.978**
Gulf sturgeon_153	0.009	0.008	0.006	**0.977**
Gulf sturgeon_154	0.004	0.005	0.005	**0.985**
Gulf sturgeon_155	0.004	0.004	0.005	**0.988**
Gulf sturgeon_156	0.006	0.005	0.005	**0.984**
Gulf sturgeon_157	0.008	0.008	0.008	**0.975**
Gulf sturgeon_158	0.005	0.005	0.004	**0.987**
Gulf sturgeon_159	0.005	0.004	0.004	**0.987**
Gulf sturgeon_160	0.005	0.005	0.004	**0.985**

Baltic_01	**0.575**	0.186	0.232	0.008
Baltic_03	**0.982**	0.008	0.005	0.005
Baltic_04	**0.943**	0.046	0.007	0.003
Baltic_05	**0.974**	0.008	0.013	0.005
Baltic_07	**0.975**	0.013	0.005	0.008
Baltic_08	**0.986**	0.005	0.004	0.005
Baltic_09	0.479	**0.492**	0.024	0.006
Baltic_10	**0.891**	0.074	0.032	0.004
Baltic_11	**0.900**	0.088	0.005	0.007
Baltic_12	**0.917**	0.012	0.006	0.064
Baltic_13	**0.979**	0.008	0.007	0.005
Baltic_14	**0.971**	0.017	0.007	0.005
Baltic_15	**0.975**	0.006	0.009	0.010
Baltic_16	**0.848**	0.14	0.005	0.007
Baltic_17	**0.971**	0.015	0.006	0.008
Baltic_18	**0.976**	0.015	0.005	0.004
Baltic_19	**0.978**	0.01	0.007	0.005
Baltic_20	**0.936**	0.014	0.043	0.007
Baltic_21	**0.902**	0.082	0.005	0.011
Baltic_22	**0.942**	0.039	0.014	0.005
Baltic_23	**0.975**	0.014	0.004	0.006
Baltic_24	**0.954**	0.019	0.022	0.005
Baltic_26	**0.969**	0.004	0.020	0.006
Baltic_27	**0.949**	0.034	0.012	0.004
Baltic_29	**0.977**	0.013	0.005	0.006
Baltic_30	**0.972**	0.009	0.015	0.004
Baltic_31	**0.983**	0.008	0.005	0.004
Baltic_32	**0.986**	0.006	0.005	0.004
Baltic_33	**0.984**	0.006	0.005	0.006

Probability of assignment values conducted in STRUCTURE based on microsatellites. Highest probabilities are listed in bold. Inferred clusters are given in Figure 1A.

**Table 3:** *Fst* statistics.

Population	1	2	3	4	5
Canadian	0	***	***	***	ns.
Mid-Atlantic	0.42	0	***	***	***
Southeast	0.38	0.15	0	***	***
Gulf of Mexico	0.65	0.41	0.27	0	***
Baltic	0.02	0.44	0.43	0.67	0

Pairwise *Fst *estimates based on partial control region sequences calculated in Arlequin v. 3.0. by computing conventional *F*-Statistics from haplotype frequencies [8,41].

**Table 4:** AMOVA.

Source of variation	d.f.	Sum of squares	Variance components		Percentage of variation
Among populations	4	78.63	0.16	Va	47.98
Within populations	625	110.19	0.18	Vb	52.02

Total	629	188.81	0.34		

Fixation	Index	*Fst*:	0.48		

Analysis of molecular variance (AMOVA) based on mitochondrial haplotype distances calculated in Arlequin v. 3.0 [8].

### Identification of hybrids

**Figure 2:**
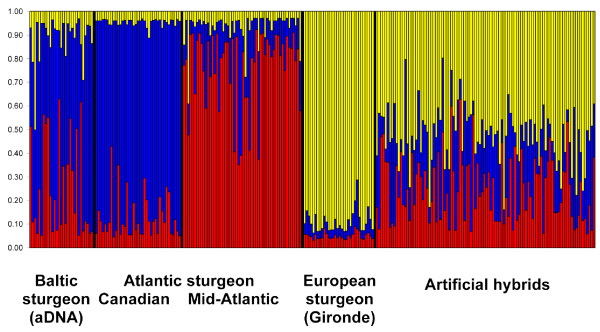
**Hybrid assignments**. Assignment test using STRUCTURE clustering Baltic founders (ancient DNA), source populations (Mid-Atlantic and Canadian sturgeons), Gironde sturgeons (*A. sturio*) and artificially generated hybrids between Gironde sturgeons and specimens from the Mid-Atlantic and Canadian source populations (different groups separated by black lines, cluster associated with colors).

Flanking sequences of locus *Aox-23 *were successfully amplified for 47 (of 50) scutes as previously described [3]. Three hybrids (fish with nuclear sequences from both species) and four introgressed specimens (mtDNA = *A. sturio *and nDNA = *A. oxyrinchus*) were identified. Additional assignment tests calculated in STRUCTURE including 100 artificial hybrids generated between fishes from source populations (Canadian, Mid-Atlantic) and European sturgeons (*A. sturio*) designed in HYBRIDLAB 1.0 clustered Baltic sturgeon together with Atlantic sturgeon, and produced no evidence for a historic hybrid population (Figure 2).

### Inference of the founder population size

Using ancient and contemporary DNA data for eight genetic loci (7 autosomal microsatellites and mtDNA), the size of the founding population to the Baltic Sea was inferred using the Approximate Bayesian Computation (ABC) method. When the posterior densities obtained for the 8 genetic loci are combined, the effective founding population size is likely to be less than 10 (Table 5, the baseline case). To evaluate the sensitivity of the results to the assumptions and methods used, different population histories, parameter values and estimation methods were tested. These included a more limited source population (Canadian only), the larger/smaller sizes of the modern North American/Baltic populations, different time points for colonization, and different rejecting/weighing procedures. Although the 95% HPD (Highest Probability Density) intervals varied, the estimated total population sizes were less than 20 individuals in most cases. A strong bottleneck signal was exhibited by both mtDNA data and also a few microsatellite loci. The assumption about the source population had a strong impact on the results. The 95% HPD interval became bigger when the Canadian population was assumed to be the only source, because the resolving power of the statistical analysis declined due to their low genetic diversity.

**Table 5:** Demographic modeling.

	Posterior density (*N*_*F*_)		Assumptions					
	Mean	95% HPD	*N*_*A*_	*N*_*B*_	*T*_*F*_	*T*_*bot*_	Source	Remark

1	3.8	2–10	2000	2000	60	1	Ca+Mid	baseline
2	3.0	2–6	10000	2000	60	1	Ca+Mid	
3	20.4	2–82	2000	2000	60	1	Ca	
4	3.6	2–10	2000	2000	50	1	Ca+Mid	
5	18.6	2–38	2000	2000	60	10	Ca+Mid	
6	10.4	2–26	2000	2000	60	---	Ca+Mid	an exponential growth from *N*_*F *_to *N*_*B*_

Estimated size of the founding population (*N*_*F*_) to the Baltic Sea at the Early Middle Ages. The ABC method was applied to 1,000,000 simulated genetic data sets (mtDNA control region and 7 microsatellite loci). The following population history was assumed as a baseline 1: a small part of the source (Canadian, Ca, and Mid-Atlantic, Mid) populations colonized the Baltic Sea at 1200 years or 60 generations before present (*T*_*F*_), experienced single-generation bottleneck (*T*_*bot*_), then the populations of both sides of the Atlantic (*N*_*A*_, *N*_*B*_) kept a constant size (effective population size = 2,000) until the Baltic population became extinct. Modified population assumptions were tested in the scenarios 2–6; 95% HPD (highest probability density) intervals are listed.

## Discussion

Restoration projects are often faced with the problem that little information is available when choosing a founder group for restorative breeding, especially when native populations became extinct many decades ago. One powerful way of obtaining more information is to analyze the genetic structure of historic populations and their relationships to extant populations [7]. Recent progress in ancient DNA analysis enables investigations of historic population structures [5,6]. This information can be used to select specimens for introduction from appropriate regional groups, taking under consideration that individuals from different environments may exhibit evolutionarily important differences in adaptively significant traits.

**Figure 3:**
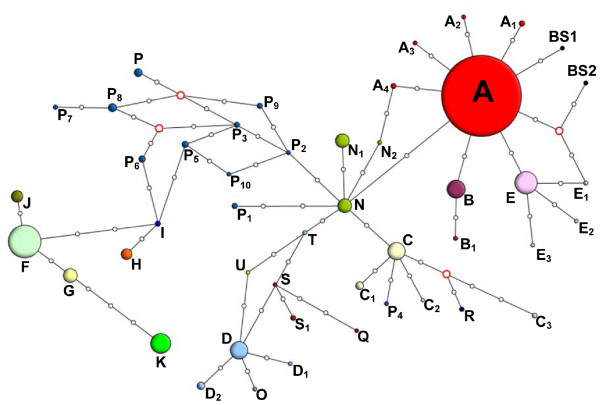
**Phylogenetic relationships of ancient and recent Atlantic sturgeon haplotypes**. Median-Joining network of American Atlantic and Baltic sturgeon haplotypes calculated in Network 4.2.0.1 based on control region sequences. Black circle white dots represent mutations and orange circle white dots represent inferred haplotypes introduced by the algorithm. Dot colors for haplotypes are congruent with colors used for mitochondrial haplotypes in figure 1.

Congruent patterns of population structuring among collections of extant *A. oxyrinchus *have been identified in both mitochondrial [9,10] and microsatellite DNA [11] which consisted of four regional clusters in the western Atlantic: 1) Gulf (*A. o. desotoi *in tributaries of the Gulf of Mexico), 2) southeastern (rivers in Georgia and South Carolina), 3) Mid-Atlantic (Hudson and Delaware rivers), and 4) Canadian (Kennebec, St. Lawrence and St. John) (Figure 1B). In the present analysis of the microsatellite profiles of the ancient Baltic population, 28 out of 29 (97%) individuals were assigned to the Canadian regional grouping and one fish was assigned to the Mid-Atlantic grouping (Figure 1A) as identified in previous studies. An overwhelming predominance of Canadian *A. oxyrinchus *genotypes within the ancient Baltic population was similarly observed in the mtDNA sequence data set (Figure 1C); 218 of 227 (96%) bony scutes shared haplotype A while the two remaining specimens had haplotypes BS1 and BS2, which are likely recent derivatives from haplotype A (Figure 3). However, it is difficult to decide when and where these "new" haplotypes evolved; prior to colonization in North America, or after the founding event in the Baltic. If there were 3 or more female founders, it is possible that BS1 and BS2 may have evolved in North America. Prior this study, 45 North American haplotypes from this control region fragment have been described. Many of them were observed in only a few fish, sometimes single specimens [e.g. [9,10,12,13]]. Considering the fact that most North American populations are representing less than 10% of their population sizes 100 years ago [14], haplotypes BS1 and BS2 could have become regionally extinct in North America. By contrast, if there was only one female founder, the two haplotypes must have evolved in the Baltic Sea.

These results demonstrate that the most northerly distributed *A. oxyrinchus *successfully colonized the Baltic Sea, suggesting that Canadian specimens may have characteristics suitable for the environmental and ecological conditions that existed during the original founding. The IUCN reintroduction guidelines state that the organisms used for reintroduction should be as closely related as possible genetically to those originally inhabiting the habitat [15]. We suggest therefore that Canadian specimens should dominate the broodstock for reintroduction.

As recent physiological and biogeographic studies implicate temperature as a primary selection force for species survival and persistence of populations [16,17], a second factor for consideration might be including specimens from populations with broader thermal tolerances in order to minimize risk to the restored population through climate change. The inclusion of specimens from the Mid-Atlantic population could potentially extend the thermal amplitude in associated physiological responses.

In any case, from an ecological point of view, there are potentially many factors which might contradict each restoration plan [18,19] (e.g. climate change, concurrence with other species, introduction of parasites or diseases).

We observed a small number of hybrids and introgressed specimens indicating a historic Baltic population of *A. sturio*; a conclusion that is supported by the archaeological record [2]. Recently, the Baltic population was suggested to be a hybrid population between European sturgeons and Atlantic sturgeons [20]. However, this conclusion is not supported by the outcome of this study. Taking genotype distribution observed for the 8^th^–13^th ^centuries [this study] and 18^th^–19^th ^centuries [3] under consideration, most likely both species were sympatric during the founding event. Later, *A. sturio *was likely displaced by Atlantic sturgeons due to the cooling during the Little Ice Age. *A. sturio *may have evolved characteristics suitable for a warmer environment [*A. sturio *needs spawning temperatures ≥ 20°C whereas spawning temperatures of *A. oxyrinchus *range between 13–26°C; reviewed in [2]] rendering the species physically unable to persist permanently in the Baltic region. Baltic region was the most eastern historic distribution area of *A. sturio*. This region is characterized by a cold, continentally influenced climate. However, immigrations (colonization attempts) of *A. sturio *from the North Sea (e.g., Elbe River) into Baltic rivers can not be excluded during warmer decades until the North Sea population became extinct during the last century.

Assuming Canadian and Mid-Atlantic populations of *A. oxyrinchus *as the original founders, our simulations suggested that the Baltic Sea was colonized by fewer than 10 founders (females and males). The estimated number of founders changed as components of the simulation model were varied, but the estimated mean was 20 individuals at the largest. This finding was based on a discrete-generation model and relatively simple population dynamics. It must be noted that the assumption of constant population size is not likely to be valid, as intensive harvest caused drastic changes to population sizes. However, testing of several different scenarios indicated that this result was quite robust. There may have been several colonization events, but the outcome of this study indicates that only one of them is likely to have succeeded. From a genetic point of view, our study suggests that it may be possible for a small number of founders to result in a sustainable population.

## Conclusion

Ancient DNA population genetic studies are a valuable tool for obtaining more information on historic population structure and information to select specimens for introduction from appropriate regional groups. Furthermore, our results indicate that only a small number of individuals may have been sufficient for the establishment and persistence of a self-sustaining population. This agrees with recent studies which suggest that successful colonization from a small number of individuals probably occurs more often than previously thought [21]. Our findings suggest that given a suitable environment, a long-term viable population may result from even a small founding population with limited genetic diversity, thus encouraging ongoing efforts to preserve and restore populations.

## Methods

### Archaeological samples

Bony scutes were excavated from two Medieval sites at the German Baltic coast, i.e. Ralswiek (Isle of Rugia, n = 538) and Wilhelmshof (Peninsula Usedom; n = 48). According to the historic record Ralswiek was a marine trading port in the late 8^th ^and 9^th ^centuries [22]. In the succeeding centuries (10^th ^– 12^th ^c.) the site lost its importance and became an agrarian settlement. Excavations (1972–1984) revealed a large faunal collection with numerous fish remains. The bony scutes of sturgeons studied here are from the early period in which sturgeons were very common and important in human diet during this time for consumption as indicated by the archaeological context [23]. In the late period (10^th ^– 12^th^c.) sturgeons are rare among the fish remains from the cultural layers, indicating a decline in sturgeon occurrence. Similar temporal changes in the importance of sturgeon as a fish for consumption have been observed at other important Medieval sites of the Baltic coast, i.e. Gdansk (Poland) and Staraja Ladoga (Russia). Wilhelmshof is a non-agrarian settlement of the 12^th^–13^th ^centuries with evidence for local handicraft and trade [24]. A small collection of fish remains (n = 178) is available from this site. Sturgeon is represented by 48 bony scutes, which were targets of the morphological and genetic analyses. Both species have different scute surfaces [25,26]. Scute surfaces of *A. oxyrinchus *are alveolar, while *A. sturio *have tubercular surfaces [drawings of scutes were published recently in [2]].

### Authenticity of DNA Sequences

DNA extraction and PCR were performed at the ancient DNA Laboratory at the Paleogenetics Group at the Institute of Anthropology of the University of Mainz, a laboratory dedicated to ancient DNA analyses following strict guidelines. We applied the criteria for the authenticity of ancient DNA as previously described [27]. DNA was extracted from bony scutes after UV irritation from each side for 30 minutes. For each scute 0.25–0.5 g material was milled and incubated overnight in 2 ml EDTA buffer, 200 μl N-Laurylsarcosidase and 20 μl Proteinase-K followed by a phenol-chlorophorm extraction with a final concentration step using Centricon^©^-100 columns. Blank controls were included in every DNA extraction as well as in every PCR. Sturgeons had never been analyzed in the ancient DNA laboratory before. No evidence for contamination was detected during the entire study.

### Mitochondrial DNA analysis

Cloning (Invitrogen) and sequencing (3100 ABI capillary sequencer; Applied Biosystems) were performed at the Leibniz Institute for Zoo and Wildlife Research, Berlin using standard procedures. PCR was performed using primers Hetero I and Hetero II or RevA, amplifying a short fragment of the control region (~200 bp) as previously described [28]. PCR products were purified by treatment with ExoSAP-IT™ (USB). A minimum of two independent PCRs were performed for each DNA extraction. Analysis of molecular variance (AMOVA) was calculated in Arlequin v. 3.0. Intraspecific relationships were calculated using NETWORK 4.2.0.1.

### Nuclear DNA analysis

Microsatellite PCR's were performed as previously described [11,29]. Length detection using 3100 ABI capillary sequencer (Applied Biosystems) were performed at the Leibniz Institute for Zoo and Wildlife Research, Berlin using standard procedures. Again, blank controls were included in every PCR setup. We used the procedure previously described [30] to minimize allelic dropout or artifacts: all loci were amplified from two independent DNA extractions. In case of differences between both runs (homozygous *vs*. heterozygous), this procedure was repeated until a sufficiently secure result was achieved otherwise the sample was discarded. Samples with ambiguous amplifications of multiple alleles were discarded for that locus. Allele length standardization between previously published data of *A. oxyrinchus *from rivers St. Lawrence and St. John (n = 39, Canadian population), Hudson and Delaware (n = 54, Mid-Atlantic), Albermarle Sound and Altamaha River (n = 37, South East), and Suwannee River (n = 48, Gulf) [11] and our ancient samples (taking into account different running conditions and devices between both labs) were performed on sample exchanges and validation of allele lengths after finishing ancient DNA analysis because shifts of +/- one allele can be found between genotyping platforms. A model-based assignment test was performed based on microsatellite data using STRUCTURE 2.0 [31]. Neither hybrids nor introgressed specimens (see below) were included in assignment tests. All 29 ancient samples included in the assignment test were classified as *A. oxyrinchus *based on their morphology and shared mtDNA *A. oxyrinchus*-haplotype A. No signs of hybridization or introgression as indicated by their microsatellite locus *Aox-23 *flanking-region sequences were observed. Each scute produced a unique multilocus genotype. Population subdivision of *A. oxyrinchus *[Canadian, Mid-Atlantic, Southeast and Gulf populations – see [11]] was investigated using the admixture model and MCMC simulations (50,000 burn-in steps followed by 100,000 replications) for different numbers of clusters (K = 2–10). For each different K, the estimates of posterior probability Pr(X|K) (simulation summary Ln P(D)) were compared [32] choosing the ΔK showing a clear peak (K = 4–5). After this, Baltic samples (aDNA) were included using the admixture model (K = 4; 100,000 burn-in steps; 1,000,000 replicates). Ten replicated runs were calculated for comparison of Ln P(D)-values and the clustering.

### Hybrid detection

*A. sturio *and *A. oxyrinchus *have several diagnostic substitutions in the flanking region of the microsatellite locus *Aox-23 *[3,29]. These substitutions were used as a hybrid marker. Hybrid detection was focused on scutes: i) showing a disagreement between morphology and mtDNA (n = 27), ii) all scutes having *A. sturio *haplotype AS17 (n = 7), and iii) to bring the sample size up to fifty we added 16 randomly selected scutes with *A. oxyrinchus *haplotype A. PCR products were cloned using the TOPO TA Cloning Kit^® ^(Invitrogen). Approximately 20 clones of each sample (n = 901 clones) were sequenced. Additionally, HYBRIDLAB 1.0 [33] was used to simulate an artificial hybrid population between *A. sturio *(Gironde population, France – allelic data were published in [34]] and *A. oxyrinchus *(Canadian population). One hundred F1-hybrid genotypes were modeled. An additional assignment test using STRUCTURE included artificially generated hybrids, potential founders (Canadian and Mid-Atlantic sturgeons), Baltic sturgeons, and Gironde sturgeons (*A. sturio*).

### Inference of the founding population size

The size of the founding population in the Baltic Sea in the Early Middle Ages was inferred from seven microsatellites and mtDNA control region sequences. The following population history was assumed in our simulations: a small part of the source (Canadian and Mid-Atlantic) populations colonized the Baltic Sea at 1,200 years before present (ybp), then the populations of both sides of the Atlantic kept a constant size (effective size = 1,000 with a 50:50 sex ratio) until the Baltic population became extinct. The Baltic founder population was assumed to experience a single-generation bottleneck, because the population size of species having a potential to produce a huge number of offspring is expected to show a dramatic increase after they settle themselves in a suitable environment. However, we also tested bottleneck periods of different lengths, as well as a gradual increase of the population size after the colonization, to check the sensitivity of the results to this assumption. Coalescent simulations were iterated 1,000,000 times, varying the effective population size of the founders as well as the source. Uniform prior distributions are assumed for both founder [1, 500] and source [100, 10,000] females as well as mutation rates (one mutation in [10,000, 100,000] years). In general, fishes are characterized by very low mutation rates and sturgeons have one of the lowest mutation rates within all vertebrates [35]. As we analyzed ancient Baltic samples (microsatellites: n = 18–30, mtDNA n = 218) and NA modern samples (microsatellites: n = 93, mtDNA n = 183) as real data, we took an equivalent number of ancient samples from the simulated Baltic population at 800 ybp as well as of modern samples from the simulated NA population. A stepwise mutation model was used for microsatellite evolution, while an infinite site model was used for mtDNA evolution. Generation time was assumed to be 20 years [36]. A discrete-generation coalescent method [37] was used to follow the change in the allele frequencies.

The approximate Bayesian computation (ABC) method [38] was applied to the simulated data set. The analyses were carried out using functions of the statistical package R provided by Mark Beaumont (University of Reading, UK). Out of the three elements (local regression, local weighing, and local density estimation) of the original ABC, the local regression procedure has a potential problem. The actual founder size used in each simulation iteration is increased or decreased by local regression on the basis of the deviation of simulated genetic data from the observed data. Because the range of founder sizes is rather small in the present study, the mathematical treatment can produce zero or negative founder sizes which never happen in the real world. Therefore, we carried out the full ABC analysis after log transformation of the variable. We also confirmed that our conclusions were unchanged if we used the untransformed data and applied the ABC without local regression to them. Posterior probability was calculated for each locus based on the following summary statistics: number of alleles, number of private alleles, and Nei's gene diversity (for both microsatellites and mtDNA); and number of segregating sites (mtDNA only). Normalized Euclidian distances between the summary statistics values of the simulated data and those of the observed data [39] were calculated for each iteration. Each locus showed a different bottleneck signal, but our main discussion was based on the combined posterior probability. One thousand out of 1,000,000 simulated data (*p*_δ _= 0.001) with the smallest distances were selected and used in the final analyses. Local weighing and calculations of the posterior density functions were carried out for each locus using the R functions.

## Abbreviations

mt: mitochondrial; a: ancient; NA: North America.

## Authors' contributions

AL initiated the study, did statistical analysis and wrote the paper. UA did experimental work. SL did statistical analysis (hybrid detection). NB provided archaeological samples and background information. LD did morphological analyses. TLK did analysis of NA populations and revised the language. SM did demographic modelling. All authors read and approved the final manuscript.

## Supplementary Material

Additional file 1 Alignment of partial d-loop sequences of *A. sturio *(As) and *A. oxyrinchus *(Ao) (haplotypes AodF-AodK were taken from Ong et al. 1996, Copeia 1996(2):464–9; no accession numbers are archived in Genbank). Click here for file
