# Supplementary material for: Tracing the first steps of American sturgeon pioneers in Europe
Source: BMC Evol Biol. 2008 Jul 29;8:221. doi: 10.1186/1471-2148-8-221 (PMC2527320; doi:10.1186/1471-2148-8-221)
Supplement: Additional file 1 — Alignment of partial d-loop sequences of A. sturio (As) and A. oxyrinchus (Ao) (haplotypes AodF-AodK were taken from Ong et al. 1996, Copeia 1996(2):464–9; no accession numbers are archived in Genbank). [file 1471-2148-8-221-S1.doc]

Additional file 1

Alignment of partial d-loop sequences of *A. sturio* (As) and *A. oxyrinchus* (Ao) (haplotypes AodF-AodK were taken from Ong et al. 1996, Copeia 1996(2):464-9; no accession numbers are archived in Genbank).

#AsG_AJ249673 TAAGATTCTACATTAAACTATTCTCTGACCACATGTCTGACCCAT---ACCAATGTCTGCATACATTAAATTGTACATACATAAACATACTATGTTTAATCCCCATTAATTTCTAGCCACCAA TACTAATGTTTACCTATATATTAAATTATCTAAGTACATAGACATACTATGTTTAATCCCCATTAATTTCTAGTCAACATATCA

#Asl7_AJ428274 TAAGATTCTACATTAAACTATTCTCTGACCACATGTCTGACCCAT---ACCAATGTCTGCATACATTAAATTGTACATACATAAACATACTATGTTTAATCCCCATTAATTTCTAGCCACCAA TACCAATGTTTACCTATATATTAAATTATCTAAGTACATAGACATACTATGTTTAATCCCCATTAATTTCTAGTCAACATATCA

#AoA_AF162716 TAAGATTCTACATTAAACTATTCTCTGGCCATATCA-TGCTCGCCTACACCAATGTTT--ATACATTAAATTGCTTATGCATGGACATATTATGTTTAATCCCCATTAATTTCTAGTCACCA-TACCAATGTTTATATATACATTAAGCCATTTAAGTACATGAACATACTATGTTTTATCCCCATTAACTTCTAGCCACTATAACA

#AoA1_AF162717 TAAGATTCTACATTAAACTATTCTCTGGCCATATCA-TGCTCGCCTACACCAATGTTT--ATACATTAAATTGCTTATGCATGGACATATTATGTTTAATCCCCATTAATTTCTAGTCACCA-TACCAATGTTTATATATACATTAAGCTATTTAAGTACATGAACATACTATGTTTTATCCCCATTAACTTCTAGCCACTATAACA

#AoA2_AF162718 TAAGATTCTACATTAAACTATTCTCTGGCCATATCA-TGCTCGCCTACACCAATGTTT--ATACATTAAATTGCTTATGCATGGACATATTATGTTTAATCCCCATTAATTTCTAGTCACCA-TACCAATGTTTATATATACATTAAGCCATTTAGGTACATGAACATACTATGTTTTATCCCCATTAACTTCTAGCCACTATAACA

#AoA3_AF162719 TAAGATTCTACATTAAACTATTCTCTGGCCATATCA-TGCTCGCCTACACCAATGTTT--ATACATTAAATTGCTTATGCATGGACATATTATGTTTAATCCCCATTAATTTCTAGTCACCA-TACCAATGTTTATATGTACATTAAGCCATTTAAGTACATGAACATACTATGTTTTATCCCCATTAACTTCTAGCCACTATAACA

#AoA4_AF162720 TAAGATTCTACATTAAACTATTCTCTGGCCATATCA-TGCTCGCCTACACCAATGTTT--ATACATTAAATTGCTTATGCATGGACATATTATGTTTAATCCCCATTAATTTCTAGTCACCA-TACCAATGTTTATACATACATTAAGCCATTTAAGTACATGAACATACTATGTTTTATCCCCATTAACTTCTAGCCACTATAACA

#AoB_AF162721 TAAGATTCTACATTAAACTATTCTCTGGCCATATCA-TGCTCGCCTACACCAATGTTT--ATACATTAAATTACTTATGCATGGACATATTATGTTTAATCCCCATTAATTTCTAGTCACCA-TACCAATGTTTATATATACATTAAGCCATTTAAGTACATGAACATACTATGTTTTATCCCCATTAACTTCTAGCCACTATAACA

#AoB1_AF162753 TAAGATTCTACATTAAACTATTCTCTGGCCATATCA-TGCTCGCCTACACCAATGTTT--ATACATTAAATTACTTATGCATGGACATATTATGTTTAATCCCCATTAATTTCTAGTCACCA-TACCAATGTTTATATATACATTAAGCCATTTAAGTACATGAACATACTATGTTTTATCCCCATTAACTTCTAGTCACTATAACA

#AoC_AF162722 TAAGATTCTACATTAAACTATTCTCTGGCCATATCA-TGCTCGCCTACACCAATGTTT--ATACATTAAATTGCTTATGCATGGACATACTATGTTTAATCCCCATTAATTTCTAGTCACCA-TACCAATGTTTATATATACATTAAACCATTTAAGTACATGAACATACTATGTTTTATCCCCATTAACTTCTAGCCACTATAACA

#AoC1_AF162723 TAAGATTCTACATTAAACTATTCTCTGGCCATATCA-TGCTCGCCTACACCAATGTTT--ATACATTAAATTGCTTATGCATGGACATACTATGTTTAATCCCCATTAATTTCTAGTCACCA-TACCAATGTTTATATATACATTAAACCATTTAAGTACATGAACATACTATGTTTTATCCCCATTAACTTTTAGCCACTATAACA

#AoC2_AF162724 TAAGATTCTACATTAAACTATTCTCTGGCCATATCA-TGCTCGCCTACACCAATGTTT--ATACATTAAATTGCTTATGCATGGACATACTATGTTTAATCCCCATTAATTTCTAGTCACCA-TACCAATGTTTATATATACATTAAACCATTTAAGTACATGAACATACTATGTTTTATCCCCATTAACTTCTAGCCAGTATAACA

#AoC3_AF162725 TAAGATTCTACATTAAACTATTCTCTGGCCATATCA-TGCTCGCCTACACCAATGTTT--ATACATTAAATTGCTTACGCATGAACATACTATGTTTAATCCCCATTAATTTCTAGTCACCA-TACCAATGTTTATATATACATTAAACCATTCAAGTACATGAACATACTATGTTTTATCCCCATTAACTTCTAGCCACTATAACA

#AoD_AF162726 TAAGATTCTACATTAAACTATTCTCTGGCCATATCA-TGCTCGCCTACACCAATGTTT--ATACATTAAATTGCTTATGCATGGACATACTATGTTTAATCCCCATTAATTTCTAGTCACCA-TATCAATGTTTATATATACATTAAGCCATTTAAGTACATGAACATACTATGTTTTATCCCCATTAACTTCTAGTCACCATAACA

#AoD1_AF162727 TAAGATTCTACATTAAACTATTCTCTGGCCATATCA-TGCTCGCCTACACCAATGTTT--ATACATTAAATTGCTTATGCATGGACATACTATGTTTAATCCCCATTAATTTCTAGTCACCA-TATCAATGTTTATATATACATTAAGCTATTTAAGTACATGAACATACTATGTTTTATCCCCATTAACTTCTAGTCACCATAACA

#AoD2_AF162728 TAAGATTCTACATTAAACTATTCTCTGGCCATATCA-TGCTCGCCTACACCAATGTTT--ATACATTAAATTGCTTATGCATGGACATACTATGTTTAATCCCCATTAATTTCTAGTCACCA-TATCAATGTTTATATATACATTAAGCCATTTAAGTACATGAACATACTATGTTTTATCCCCATTAACTTTTAGTCACCATAACA

#AoE_AF162729 TAAGATTCTACATTAAACTATTCTCTGGCCATATCA-TGCTCGCCTACACCAATGTTT--ACACATTAAATTGCTTATGCATGGACATATTATGTTTAATCCCCATTAATTTCTAGTCACCA-TACCAATGTTTATATATACATTAAGCCATTTAAGTACATGAACATACTATGTTTTATCCCCATTAACTTCTAGCCACTATAACA

#AoE1_AF162730 TAAGATTCTACATTAAACTATTCTCTGGCCATATCA-TGCTCGCCTACACCAATGTTT--ACACATTAAATTGCTTATGCATGGACATATTATGTTTAATCCCCATTAATTTCTAGTCACCA-TACCAATGTTTATATATACATTAAGCCATTTAAGTACATGAACATACTATGTTTTGTCCCCATTAACTTCTAGCCACTATAACA

#AoE2_AF162731 TAAGATTCTACATTAAACTATTCTCTGGCCATATCA-TGCTCGCCTACACCAATGTTT--ACACATTAAATTGCTTATGCATGGACATATTATGTTTAATCCCCATTAATTTCTAGTCACCA-TACCAATGTTTACATATACATTAAGCCATTTAAGTACATGAACATACTATGTTTTATCCCCATTAACTTCTAGCCACTATAACA

#AoE3_AF162749 TAAGATTCTACATTAAACTATTCTCTGGCCATATCA-TGCTCGCCTACACCAATGTTT--ACACATTAAATTGCTTATGCATGGACATATTATGTTTAATCCCCATTAATTTCTAGTCACCA-TACCAATGTTTATATATACATTAAGCCATTTAAGTACATGAACATACTATGTTTTATCCCCATTAACTTCTAGTCACTATAACA

#AoN_AF162732 TAAGATTCTACATTAAACTATTCTCTGGCCATATCA-TGCTCGCCTACACCAATGTTT--ATACATTAAATTGCTTATGCATGGACATACTATGTTTAATCCCCATTAATTTCTAGTCACCA-TACCAATGTTTATATATACATTAAGCCATTTAAGTACATGAACATACTATGTTTTATCCCCATTAACTTCTAGCCACTATAACA

#AoN1_AF162733 TAAGATTCTACATTAAACTATTCTCTGGCCATATCA-TGCTCGCCTACACCAATGTTT--ATACATTAAATTGCTTATGCATGGACATACTATGTTTAATCCCCATTAATTTCTAGTCACCA-TACCAATGCTTATATATACATTAAGCCATTTAAGTACATGAACATACTATGTTTTATCCCCATTAACTTCTAGCCACTATAACA

#AoN2_AF162734 TAAGATTCTACATTAAACTATTCTCTGGCCATATCA-TGCTCGCCTACACCAATGTTT--ATACATTAAATTGCTTATGCATGGACATACTATGTTTAATCCCCATTAATTTCTAGTCACCA-TACCAATGTTTATACATACATTAAGCCATTTAAGTACATGAACATACTATGTTTTATCCCCATTAACTTCTAGCCACTATAACA

#AoO_AF162735 TAAGATTCTACATTAAACTATTCTCTGGCCATATCA-TGCTCGCCTACACCAATGTTT--ATACATTAAATTGCTTATGCATGGACATACTATGTTTAATCCCCATTAATTTCTAGTCACCA-TATCAATGTTTGTATATACATTAAGCCATTTAAGTACATGAACATACTATGTTTTATCCCCATTAACTTCTAGTCACCATAACA

#AoP_AF162736 TAAGATTCTACATTAAACTATTCTCTGGCCATATCA-TGCTCGCCTACACCAATGTTT--ATACATTAAATTGCTTATACATGGACATACTATGTTTAATCCCCATTAATTTCTAGTCACCA-TACCAATGCTTATATATACATTAAGCCATTTAAGTACATGAACATGCTATGTTTTATCCTCATTAACTTCTAGCCACTATAACA

#AoP1_AF162737 TAAGATTCTACATTAAACTATTCTCTGGCCATATCA-TGCTCGCCTACACCAATGTTT--ATACATTAAATTGCTTATGCATGGACATACTATGTTTAATCCCCATTAATTTTTAGTCACCA-TACCAATGTTTATATATACATTAAGCCATTTAAGTACATGAACATATTATGTTTTATCCCCATTAACTTCTAGCCACTATAACA

#AoP2_AF162738 TAAGATTCTACATTAAACTATTCTCTGGCCATATCA-TGCTCGCCTACACCAATGTTT--ATACATTAAATTGCTTATGCATGGACATACTATGTTTAATCCCCATTAATTTCTAGTCACCA-TACCAATGTTTATATATACATTAAGCCATTTAAGTACATGAACATGCTATGTTTTATCCCCATTAACTTCTAGCCACTATAACA

#AoP3_AF162739 TAAGATTCTACATTAAACTATTCTCTGGCCATATCA-TGCTCGCCTACACCAATGTTT--ATACATTAAATTGCTTATACATGGACATACTATGTTTAATCCCCATTAATTTCTAGTCACCA-TACCAATGTTTATATATACATTAAGCCATTTAAGTACATGAACATGCTATGTTTTATCCCCATTAACTTCTAGCCACTATAACA

#AoP4_AF162740 TAAGATTCTACATTAAACTATTCTCTGGCCATATCA-TGCTCGCCTACACCAATGTTT--ATACATTAAATTGCTTATACATGGACATACTATGTTTAATCCCCATTAATTTCTAGTCACCA-TACCAATGTTTATATATACATTAAACCATTTAAGTACATGAACATACTATGTTTTATCCCCATTAACTTCTAGCCACTATAACA

#AoP5_AF162741 TAAGATTCTACATTAAACTATTCTCTGGCCATATCA-TGCTCGCCTACACCAATGTTT--ATACATTAAATTGCTTATACATGGACATACTATGTTTAATCCCCATTAATTTCTAGTCACCA-TACCAATGTTTATATATACATTAAGCCATTTAAGTACATGAACATGCTATGTTTTATCCCCATTAACTTCTAGTCACTATAACA

#AoP6_AF162742 TAAGATTCTACATTAAACTATTCTCTGGCCATATCA-TGCTCGCCTACACCAATGTTT--ATACATTAAATTGCTTATACATGGACATACTATGTTTAATCCCCATTAATTTCTAGTCACCA-TACCAATGTTTATATATACATTAAGCCATTTAAGTACATGAACATGCTATGTTTTGTCCCCATTAACTTCTAGTCACTATAACA

#AoP7_AF162743 TAAGATTCTACATTAAACTATTCTCTGGCCATATCA-TGCTCGCCTACACCAATGTTT--ATACATTAAATTGCTTATACATGGACATACTATGTTTAATCCCCATTAATTTCTAGTCACCA-TACCAATGTTTATATATACATTAAGCCATTTAAGTACATGAACATGCTATGTTCTGTCCTCATTAACTTCTAGCCACTATAACA

#AoP8_AF162744 TAAGATTCTACATTAAACTATTCTCTGGCCATATCA-TGCTCGCCTACACCAATGTTT--ATACATTAAATTGCTTATACATGGACATACTATGTTTAATCCCCATTAATTTCTAGTCACCA-TACCAATGTTTATATATACATTAAGCCATTTAAGTACATGAACATGCTATGTTTTGTCCTCATTAACTTCTAGCCACTATAACA

#AoP9_AF162745 TAAGATTCTACATTAAACTATTCTCTGGCCATATCA-TGCTCGCCTACACCAATGTTT--ATACATTAAATTGCTTATGCATGGACATACTATGTTTAATCCCCATTAATTTCTAGTCACCA-TACCAATGTTTATATATACATTAAGCCATTTAAGTACATGAACATGCTATGTTTTATCCTCATTAACTTCTAGCCACTATAACA

#AoP10_AF162746 TAAGATTCTACATTAAACTATTCTCTGGCCATATCA-TGCTCGCCTACACCAATGTTT--ATACATTAAATTGCTTATGCATGGACATACTATGTTTAATCCCCATTAATTTCTAGTCACCA-TACCAATGTTTATATATACATTAAGCCATTTAAGTACATGAACATGCTATGTTTTATCCCCATTAACTTCTAGTCACTATAACA

#AoQ_AF162747 TAAGATTCTACATTAAACTATTCTCTGGCCATATCA-TGCTCGCCTACACCAATGTTT--ATACATTAAATTGCTTATGCATGGACATACTATGTTTAATCCCCATTAATTTCTAGTCACCA-TATCAATGTTTATATATACATTAAGCCATCTAAGTACATGAACATACTATGTTTTATCCTCATTAACTTCTAGCCACCATAACA

#AoR_AF162748 TAAGATTCTACATTAAACTATTCTCTGGCCATATCA-TGCTCGCCTACACCAATGTTT--ATACATTAAATTACTTATGCATGGACATACTATGTTTAATCCCCATTAATTTCTAGTCACCA-TACCAATGTTTATATATACATTAAACCATTCAAGTACATGAACATACTATGTTTTATCCCCATTAACTTCTAGCCACTATAACA

#AoS_AF162750 TAAGATTCTACATTAAACTATTCTCTGGCCATATCA-TGCTCGCCTACACCAATGTTT--ATACATTAAATTGCTTATGCATGGACATACTATGTTTAATCCCCATTAATTTCTAGTCACCA-TATCAATGTTTATATATACATTAAGCCATTTAAGTACATGAACATACTATGTTTTATCCCCATTAACTTCTAGCCACCATAACA

#AoS1_AF162751 TAAGATTCTACATTAAACTATTCTCTGGCCATATCA-TGCTCGCCTACACCAATGTTT--ATACATTAAATTGCTTATGCATGGACATACTATGTTTAATCCCCATTAATTTCTAGTCACCA-TATCAATGTTTATATATACATTAAGCCATTTAAGTACATGAACATACTATGTTTTATCCCCATTAACTTCCAGCCACCATAACA

#AoT_AF162752 TAAGATTCTACATTAAACTATTCTCTGGCCATATCA-TGCTCGCCTACACCAATGTTT--ATACATTAAATTGCTTATGCATGGACATACTATGTTTAATCCCCATTAATTTCTAGTCACCA-TACCAATGTTTATATATACATTAAGCCATTTAAGTACATGAACATACTATGTTTTATCCCCATTAACTTCTAGCCACCATAACA

#AoU_AF162754 TAAGATTCTACATTAAACTATTCTCTGGCCATATCA-TGCTCGCCTACACCAATGTTT--ATACATTAAATTGCTTATGCATGGACATACTATGTTTAATCCCCATTAATTTCTAGTCACCA-TACCAATGTTTATATATACATTAAGCCATTTAAGTACATGAACATACTATGTTTTATCCCCATTAACTTCTAGTCACCATAACA

#AoBS1_EU684143 TAAGATTCTACATTAAACTATTCTCTGGCCATATCA-TGCTCGCCTACACCAATGTTT--ATACATTAAATTGCTTATGCATGGACATATTATGTTTAATCCTCATTAATTTCTAGTCACCA-TACCAATGTTTATATATACATTAAGCCATTTAAGTACATGAACATACTATGTTTTATCCCCATTAACTTCTAGCCACTATAACA

#AoBS2_EU684144 TAAGATTCTACATTAAACTATTCTCTGGCCATATCA-TGCTCGCCTACACCAATGTTT--ATACATTAAATTGCTTATGCATGGACATATTATGTTTAATCCCCATTAATTTCTAGTCACCA-TACCAATGTTTATATATACATTAAGCCATTTAAGTACATGAACATATTATGTTTTGTCCCCATTAACTTCTAGCCACTATAACA

#AodF TAAGATTCTACATTAAACTATTCTCTGGCCATATCA-TGCTCGCCTACACCAATGTTT--ATACATTAAATTGCTTATACATGGACATACTATGTTTAATCCCCATTAATTTCTAGTCACCA-TACCAATGTTTATATATACATTAACCCATTTAAGTACATGAACATGTTATGTTTTATCCTCATTAACTTCTAGTCACTATAACA

#AodG TAAGATTCTACATTAAACTATTCTCTGGCCATATCA-TGCTCGCCTACACCAATGTTT--ATACATTAAATTGCTTATACATGGACATACTATGTTTAATCCCCATTAATTTCTAGTCACCA-TACCAATGTTTATATATACATTAACCCATTTAAGTACATAAACATGTTATGTTTTATCCTCATTAACTTCTAGTCACTATAACA

#AodH TAAGATTCTACATTAAACTATTCTCTGGCCATATCA-TGCTCGCCTACACCAATGTTT--ATACATTAAATTGCTTATACATGGACATATTATGTTTAATCCCCATTAATTTCTAGTCACCA-TACCAATGTTTATATATACATTAAGCCATTTAAGTACATGAACATGTTATGTTTTATCCCCATTAACTTCTAGTCACTATAACA

#AodI TAAGATTCTACATTAAACTATTCTCTGGCCATATCA-TGCTCGCCTACACCAATGTTT--ATACATTAAATTGCTTATACATGGACATACTATGTTTAATCCCCATTAATTTCTAGTCACCA-TACCAATGTTTATATATACATTAAGCCATTTAAGTACATGAACATGTTATGTTTTATCCCCATTAACTTCTAGTCACTATAACA

#AodJ TAAGATTCTACATTAAACTATTCTCTGGCCATATCA-TGCTCGCCTACACCAATGTTT--ATACATTAAATTGCTTATACATGGACATACTATGTTTAATCCCCATTAATTTCTAGTCACCA-TACCAATGTTTATATATACATTAACCCATTTAAGTACATGAACATGTTATGTTTTATCCTCATTAACTTCTAGTCACCATAACA

#AodK TAAGATTCTACATTAAACTATTCTCTGGCCATATCA-TGCTCGCCTACACCAATGTTT--ATACATTAAATTGCTTATACATGGACATACTATGTTTAATCCCCATTAATTTCTAGTCACCA-TACCAATGTTTATATATGCATTAAGCCATTTAAGTACATAAACATGTTATGTTTTATCCTCATTAATTTCTAGTCACTATAACA
